# Leukemogenesis occurs in a microenvironment enriched by extracellular microvesicles/exosomes: recent discoveries and questions to be answered

**DOI:** 10.1038/s41375-024-02188-9

**Published:** 2024-02-22

**Authors:** Mariusz Z. Ratajczak, Janina Ratajczak

**Affiliations:** 1 Stem Cell Institute at Brown Cancer Center, University of Louisville, Louisville, KY USA; 2 Department of Regenerative Medicine, Center for Preclinical Research and Technology, Medical University of Warsaw, Warsaw, Poland

**Keywords:** Stem cells, Cell biology

## Abstract

In single-cell organisms, extracellular microvesicles (ExMVs) were one of the first cell-cell communication platforms that emerged very early during evolution. Multicellular organisms subsequently adapted this mechanism. Evidence indicates that all types of cells secrete these small circular structures surrounded by a lipid membrane that may be encrusted by ligands and receptors interacting with target cells and harboring inside a cargo comprising RNA species, proteins, bioactive lipids, signaling nucleotides, and even entire organelles “hijacked” from the cells of origin. ExMVs are secreted by normal cells and at higher levels by malignant cells, and there are some differences in their cargo. On the one hand, ExMVs secreted from malignant cells interact with cells in the microenvironment, and in return, they are exposed by a “two-way mechanism” to ExMVs secreted by non-leukemic cells. Therefore, leukemogenesis occurs and progresses in ExMVs enriched microenvironments, and this biological fact has pathologic, diagnostic, and therapeutic implications. We are still trying to decipher this intriguing cell-cell communication language better. We will present a current point of view on this topic and review some selected most recent discoveries and papers.

## Introduction

To get a complete insight into the mechanism that governs the biology of multicellular organisms, we need to go back in evolution and elucidate cell-cell communication pathways operating at the cell level in unicellular forms of life. One such communication platform was the “language” of extracellular microvesicles (ExMVs) [1–3]. It was probably the first means for cells to signal their presence and pass information to other cells in a surrounding microenvironment.

It has been more than twenty years since we proposed that cell-derived extracellular microvesicles (ExMVs) may transfer from one cell to another mRNA and proteins to change their biological function and phenotype [4]. This opened a new area of investigation on the paracrine role of ExMVs in several biological systems, including hematopoiesis and leukemogenesis. After an initial wave of skepticism, this fundamental cell-cell communication mechanism that originated early during the evolution of living organisms has been confirmed by other investigators, and several outstanding seminal papers on this topic have been published [5–7]. It is the best example of how sometimes novel concepts in science need time to overcome initial disbelief before becoming embraced by the research community.

ExMVs were first discovered by microscope in centrifuged blood plasma or supernatants from the cell cultures and interpreted as a kind of “cellular dust” [8–10]. Their origin was initially associated with the platelets as “platelet dust” that has been identified to provide pro-coagulation activity [11, 12]. Nevertheless, it took some time to understand the biological involvement of ExMVs secreted by other cells in several physiological and pathological processes.

The seminal discovery that ExMVs can transfer their cargo to the other cells and affect their biology, which has been published in *Leukemia* [4], opened a new area of investigation that resulted in the exponentially growing number of published papers on this topic, the founding of an International Society for Extracellular Vesicles (ISEV), and the establishment of a scientific journal devoted to the field, the Journal of Extracellular Vesicles. The creation of companies paralleled this, and funding programs focused on dissecting the biological role of ExMVs, their role as biomarkers and diagnostic tools, and their application as potential therapeutics [1, 8].

## ExMVs as modulators of cell function

**Fig. 1:**
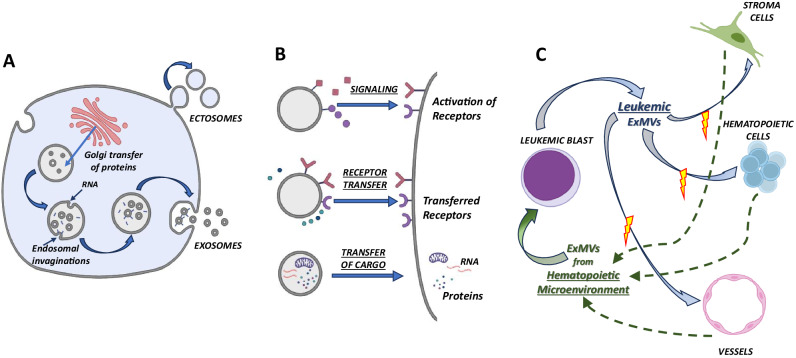
Biological effects of ExMVs. **A Cellular sources of ExMVs**. While larger ExMVs (100–1000 nm in diameter), sometimes described as ectosomes, are derived by cell-surface membrane blebbing, smaller ones, known as exosomes (50–150 nm), are derived by the multivesicular body (MVB) pathway or the Golgi apparatus pathway for exocytosis. Leukemia cells secrete more ExMVs than normal hematopoietic cells. **B** **Biological effects of ExMVs**. ExMVs may interact with receptors expressed on target cells by surface-expressed ligands (upper panel), transferring receptors to the target cells (middle panel), or transferring cargo containing mRNA, miRNA, proteins, or other biomolecules from one cell to another (lower panel). **C** **ExMVs crosstalk in leukemia cell microenvironment**. Leukemia cells secrete ExMVs that affect stroma cells, endothelial cells, and other normal hematopoietic cells. In return, these cells secrete ExMVs that affect the progression of leukemia.

Depending on their origin from the membrane compartments - (i) cell surface membrane or (ii) intracellular membranes and their size these intriguing spheric structures are called different names [1–3, 13, 14]. ExMs that are larger and measure up to 1000 nm in diameter, derived by the outward budding of cell surface membrane, are sometimes called ectosomes (Fig. 1A). In contrast, the smaller ones up to 200 nm in diameter are called exosomes. They originate by inward pinching off from the endosomal invaginations into intracellular vesicles enriched by cytosol-derived molecules called the multivesicular body (MVB), forming intraluminal vesicles. The content of MVB is subsequently released into extracellular space after their fusion with the plasma membrane (Fig. 1A). While the larger ExMVs derived by cell membrane budding express CD40, selectins, integrins, cellular receptors, and cytoskeletal proteins, and their membranes are highly enriched in cholesterol, phosphatidylserine, and diacylglycerol, small ExMvs express specific markers, such as the tetraspanin family of proteins (e.g., CD63/CD9), thermal shock proteins (HSP70/90), and major histocompatibility class I antigens [8, 15–17].

ExMVs have a negative ζ surface potential that indicates colloidal stability and allows their interaction with positively charged molecules. Due to this charge, ExMVs interact with soluble interacting partner molecules in the extracellular environment. These interacting partners include enzymes, DNA/RNA binding proteins, DNA, RNA, annexin, albumin, and coagulation cascade components. This influences ExMVs size, mobility, and distribution in biological fluids. These surface molecules form a “coronal layer” and provide a large, interactive, highly dynamic surface area that facilitates interaction with target cells [15, 18, 19]. The ζ surface potential could be affected by changes in pH or ion concentration. Overall, ions with higher valency (Ca2^+^) decrease ζ surface potential compared to monovalent ones (Na^+^, K^+^). Thus, molecules’ charge and hydrophobicity impact ExMV’s preference to bind to the target cells.

Their primary biological role as physiological nano-carriers is horizontal transfer to the target cells of functional RNA species, DNA fragments, proteins, bioactive lipids, signaling purines, nutrients, and even small organelles such as mitochondria [1–7, 16]. They can also act as “signaling devices” by stimulating neighboring cells with surface-expressed ligands [1–7]. Finally, they can transfer “hijacked” surface receptors between cells [8, 20, 21]. To summarize these biological effects (Fig. 1B), ExMVs may be considered as (i) signaling platforms when they stimulate cells with ligands embedded in their outer lipid layer, (ii) cell-surface phenotype “modifiers” if they transfer cell membrane receptors from other cells, and finally (iii) cargo-delivery packets that transfer bioactive compounds, or genetic material to the target cells. Thus, due to their unique role in exchanging material and transmitting information to the cells, ExMvs emerge as essential modulators of the biological function of other cells.

With time, other types of ExVs with different biological functions, such as “trash disposal vesicles” eliminating unwanted materials in the form of apoptotic bodies during the apoptotic death of cells, have also been described [22]. There are also identified larger oncosomes and migrasomoes involved in creating metastatic niches for cancer cells and pathfinding migrating cells through the environment [23]. These latter play an essential role in the metastasis of solid cancer cells.

The currently available experimental strategies to characterize ExMVs include nanoparticle tracking analysis that calculates size distributions and numbers of ExMVs in suspension, electron microscopy-based approaches to visualize their structure, and flow cytometry combined with antibodies against selected surface markers [8, 24–27]. Other strategies for studying molecular signatures of ExMVs are based on analysis of their cargo (mRNA species, protein, bioactive lipid content, and metabolites) by employing “omics” technologies aimed at the detection of mRNA species (transcriptomics), proteins (proteomics), lipids (lipidomics), and metabolites (metabolomics) [8, 19, 24–27]. In addition, evidence has accumulated that ExMVs also contain several extracellular signaling nucleotides - for example, adenosine triphosphate (ATP) - involved in purinergic signaling.

Several papers demonstrated that both larger (microsomes) and smaller (ectosomes) species of ExMVs play an essential role in normal hematopoiesis [4, 18, 28–31]. The hematopoietic microenvironment is enriched for these small vesical structures whose role may somehow resemble a biblical “Tower of Babel” where mutual communication became confused by the presence of many languages. Nevertheless, we are making progress in uncovering the “communication skills” of ExMVs. By analogy to physiological hematopoiesis, we learned that ExMVs also play an essential role in leukemia, and we will focus on this topic in this review. Briefly, ExMVs can affect the proliferation and survival of leukemic cells, modulate the hematopoietic microenvironment to support leukemia cell expansion, contribute to immune escape by malignant cells, and promote chemotherapy resistance [12, 24–27, 30, 31]. On the other hand, they could play a role in diagnosing hematopoietic malignancies as they express potential biomarkers and even be modified as drug carriers for therapy [22, 32–34]. This will be discussed later in this review.

## Leukemia cells are surrounded in the bone marrow microenvironment, peripheral blood, and lymph by “ExMVs dust.”

ExMVs are secreted under steady-state conditions by all hematopoietic cells, increasing their number in pathological conditions [34–38]. It is well known that leukemia cells, like other malignant cells, tend to secrete more ExMVs. As a result of this, cancer patients have a higher concentration of these circulating nano-carriers in vital body fluids, including peripheral blood (PB), lymph, and intercellular microenvironment in hematopoietic organs, including bone marrow (BM) [34–38]. ExMVs can serve as biomarkers and diagnostic tools. They can also transfer oncogenic fusion transcripts into target cells such as, for example, BCR-ABL1, TEL-AML1, and MLL-AF6 and, in addition, mRNA species related to basic functions of leukemic cells [39]. They can also transfer as reported fusion transcript characteristics for ALL cells, e.g., t(12:21, t 1:19) [39]. Moreover, in T-ALL, MVs contain NOTCH1-dependent microRNAs, which control oncogenic pathways acting as autocrine stimuli and ultimately promote the expansion/survival of highly proliferative cell subsets of human T-cell leukemias. These miRNAs mostly comprised members of the miR-17-92a cluster and paralogues regulate a network of genes in patients with relapsed/refractory early T-cell progenitor in ALL. These ExMVs enriched for NOTCH1-associated miRNAs alter the cell heterogeneity and the dynamics of T-cell leukemias [40]. Interestingly, the cargo of ExMVs may also change after chemotherapy. For example, AML cells, in response to cytarabine or decitabine, carry and transfer 3-hydroxy-3-methyl-glutaryl-coenzyme A reductase, the rate-limiting enzyme for cholesterol synthesis, and thus enhance cholesterol level in the target cells [41].

All the communication mechanisms by ExMV seen in normal cells also involve leukemia. Overall, they may regulate an unwanted progression or even potentially the initiation of blood cancer [42]. This latter effect, however, needs more direct evidence. Nevertheless, recently, an interesting hypothesis has been postulated to explain the development of donor-derived de novo hematologic neoplasm after allogeneic cell transplantation, which could be caused after transfer to transplanted donor cells recipient-derived primary neoplasm ExMVs cargo and mitochondria that may persist in patients after conditioning for transplantation [43]. This mechanism, as postulated, could be responsible for donor cells’ derived hematologic neoplasms. To support this, in another recent paper, it has been proposed that the transfer of DNA-methyltransferase-1 (DNMT1) mRNA transcript by leukemic ExMVs may reprogram normal hematopoietic cells [44].

Among all leukemia, in particular, the progression of AML is the best-studied hematological malignancy [34, 35, 37, 45]. Progress of this disorder is affected by (i) ExMVs direct signaling and (ii) phenotype modification. Secretion of ExMVs by leukemic stem cells affects non-malignant cells in the hematopoietic microenvironment and other leukemia cells. Moreover, as shown in Fig. 1C, normal cells residing in hematopoietic tissues respond to leukemic cells by secretion of their ExMVs, which may affect the proliferation and survival of malignant cells. As a result, leukemic cells are surrounded by “ExMVs dust,” collecting different information and undergoing epigenetic modification.

Several published papers have attempted to fractionate ExMVs according to their size, which may indicate their origin due to cell membrane budding or release by exocytosis from the intracellular membrane compartment. These experimental approaches yield essential information; however, we should study the biological effect of unseparated ExMVs fractions, as different biological effects may depend on their cellular origin [8, 22]. Moreover, leukemia cells are exposed to ExMVs with diverse biological effects depending on which cells in the hematopoietic microenvironment they originate from. These effects are mediated by surface-expressed ligands or the content of fused or internalized inside cargo. Accordingly, ExMVs may (i) interact with cell surface receptors, (ii) fuse with target cells to release their cargo into the cytosol, or (iii) they can be internalized [8, 27, 29, 30, 33, 34, 45]. The first two mechanisms are the most important, as internalization may lead to lysosomal degradation of ExMVs content. However, uptake by internalization can still provide some nutrients and metabolites to the cells.

Unsurprisingly, leukemic ExMVs display different molecular profiles than ExMVs derived from normal hematopoietic cells [24–27]. The molecular signature of leukemic ExMVs varies, depending on the type of hematological malignancy and the stage of the disease [24]. These differences are observed both in the expression of some surface markers and the composition of their inner content. The molecular signature of ExMVs may also be affected by several other factors that need better characterization, such as age and sex of the patient, co-existing disorders, diet, and circadian rhythm state of cells releasing them to the microenvironment. Moreover, ExMVs surrounding leukemic cells can also exert opposite pleiotropic effects. Table 1 summarizes several possibilities for how ExMVs may affect the initiation and progression of leukemia. Since ExMVs could be a clinical problem, several strategies have been proposed to eliminate them from circulation [8, 17, 34, 45]. This will be discussed later by addressing the pros and cons of such developmental therapeutic approaches.

**Table 1:** Examples of biological effects of ExMVs in leukemia.

Biological effects of ExMVs in leukemia	Selected references
Remodeling hematopoietic microenvironment and stem cell niches, transforming BM stroma to leukemia-supporting cells	[24, 27, 35, 54]
Maintenance of stemness of leukemia cells	[24, 39, 40]
Providing resistance of leukemic cells to apoptosis and cell senescence	[17, 24, 34, 36, 46]
Suppression of normal hematopoiesis	[17, 24, 41]
Promoting leukemia cells’ drug resistance	[24, 58, 59]
Stimulating angiogenesis	[17, 24, 44]
Providing immunosuppression to allow escape of leukemic cells’ immune response	[24, 49, 55, 56]
Modulation of leukemic cell metabolism	[17, 24, 33, 50, 52]

## Leukemia cell-derived ExMVs and their interaction with hematopoietic microenvironment

The hematopoietic microenvironment is the home of normal hematopoietic stem cells (HSCs), facilitating their specification into progenitor cells and expansion into differentiated cells from all hematopoietic lineages. It also contains many other cells, such as fibroblasts, endothelial cells, and cells lining bone niches, providing signals supporting hematopoiesis. Evidence has accumulated that leukemia cell-derived ExMVs in BM remodel the microenvironment to favor their expansion [27, 34, 41, 45]. Accordingly, they modify BM hematopoietic stem cell niches, stimulate angiogenesis, and transform BM stroma cells into leukemia-supporting cells. This involves all types of interaction between ExMVs and target cells depicted in Fig. 1B. As a result of this, it is perturbed, for example, proper retention of normal HSCs in BM niches due to the (i) downregulation of, e.g., stromal-derived factor-1 (SDF-1), kit ligand (KL) and (ii) remodeling of intercellular matrix [27, 29, 45]. This leads to the release of HSCs from their niches. ExMVs may also directly inhibit HSC expression of BM retention and survival receptors, such as the CXCR4 receptor for SDF-1 and the c-kit receptor for KL [27, 29, 45]. Some miRNA species responsible for suppressing normal hematopoiesis have been identified by interference with the expression of pro-survival pathways directly in HSCs or due to modulating the expression of HSCs-promoting factors in the BM microenvironment [27, 29, 41, 45, 46]. It has been reported, for example, that AML cells secrete exosomes enriched in miR-150 and miR-155 that impair normal hematopoiesis by inhibiting the expression of c-MYB [34, 41]. Besides miRNA, other components of ExMVs are involved in these effects, including proteins and enzymes. It is known that leukemia-cell-derived ExMVs induce inflammation in the BM microenvironment and promote suppression of normal hematopoiesis due to the secretion of several pro-inflammatory cytokines from the stroma and endothelium [41, 46]. Recently, it has been also postulated an additional pro-inflammatory mechanism by which normal HSCs become recruited by leukemia ExMVs to adopt pro-inflammatory phenotype, and this pro-inflammatory conversion in long-lived HSCs in the BM along with their regenerative re-expansion during remission may impact clonal selection of leukemia cells and disease evolution [29]. Moreover, as reported, ExMVs derived from myelodysplastic stromal cells may also induce DNA damage and mutagenesis in normal hematopoietic cells through miRNA transfer [46]. Recently, long non-coding RNAs (lncRNAs) have been shown to regulate gene expression and display cross-talk with various miRNAs species. As proposed, lncRNAs exert “sponge-like” effects to capture miRNAs and may modulate miRNA-mediated functions. This crosstalk between two types of RNAs may contribute to the pathogenesis of some diseases [47].

An essential effect of leukemia cell-derived ExMVs is their ability to stimulate angiogenesis and expansion of fibroblasts in BM microenvironments [34–36, 48]. Stimulated endothelial cells and stroma fibroblasts secrete several trophic factors that will be discussed in the next paragraph. These factors may promote the expansion of leukemic cells and increase their survival and resistance to chemotherapy. Similarly, leukemia-derived ExMVs may also transform monocytes into “tumor-associated macrophages” that secrete several factors that promote the proliferation and survival of cancer cells [49].

## ExMVs in propagating survival and proliferation of leukemic cells

Evidence has accumulated that leukemia cells with stem cell phenotype may secrete ExMVs that promote proliferation, survival, and chemoresistance of other malignant cells, creating a positive self-perpetuating signaling loop. This role has been identified for example for fibroblast growth factor-2 (FGF2), or HMG-CoA reductase, which is the rate-limiting enzyme for cholesterol synthesis, increasing after transfer cholesterol synthesis in leukemic cells to provide material for the formation of cell membranes in rapidly growing cells [50]. There are also involved in these effects some miRNA species, e.g., miRNA-1246, that activate the STAT3 pathway in leukemic cells [51].

On the other hand, an important role play ExMVs secreted by BM-residing fibroblasts and endothelial cells in response to the presence of “intruders” that are leukemia cells in the hematopoietic microenvironment [36]. These ExMVs are enriched in FGF2, TGFβ-1, Angiopoietin-related protein 2, and again in several miRNA species that may stimulate proliferation, resistance to apoptosis, and chemotherapy of leukemic blasts [45]. In addition to stroma-, endothelial cells- and other hematopoietic cells-derived ExMVs, the growth of leukemic cells could be also modified by platelet-derived ExMVs [52]. They can not only decorate tumor cells with “platelet-hijacked” receptors important for interaction with endothelium [8] but, as recently reported, reprogram metabolically chronic lymphocytic leukemia (CLL) cells. In this effect are involved so-called mito-ExMVs that are highly enriched for mitochondria that, after internalization due to enhanced *oxidative phosphorylation* (OXPHOS), lead to metabolic rewiring of tumor cells and provide them with additional resistance to chemotherapy [52]. Similarly, as reported recently, MSCs-derived ExMVs may rescue chronic lymphocytic (CLL) B cells from apoptosis and induce gene expression modification by increasing the expression of CCL3/4 and EGR1/2/3 pathways [46].

However, the pro-leukemic effects of ExMVs have been reported in several seminal papers; there are also some red lights for opposite effects, and for example, MSCs-ExMVs cells decreased the chemosensitivity of ALL blasts by transferring miR-10a [53]. Similarly, in recent experiments with leukemic cell lines, MSCs-derived ExMVs increased the expression of BID and BAX and decreased the expression of BCL2, indicating induction of apoptosis [36]. These are examples of how the “communication language” of ExMVs is complicated, and some effects could be different or even opposite than initially expected.

## ExMVs suppress the immune response against leukemia cells and enhance resistance to chemotherapy

An essential aspect of leukemia-promoting expansion by ExMVs is their role in damping immune reactions against leukemic blasts and inducing resistance to chemotherapy. The amelioration of immune response could be related to ExMVs mediated (i) inhibition of cells involved in anti-leukemic effects such as natural killer (NK) cells and CD8^+^T lymphocytes, and (ii) activation in hematopoietic organs immunosuppressive cells including regulatory T cells (Tregs) and myeloid-derived suppressor cells (MDCs) [54].

Several mechanisms involved in the induction of immunosuppression are described to illustrate this. It has been reported, for example, that ExMVs carrying death ligands may induce apoptosis of CD8^+^T cells [55]. On the other hand, leukemic ExMVs may lower the expression of NKG2D on NK cells and impair their antitumor activity [56]. Moreover, the antitumor effect of Tregs could be affected by ExMVs-mediated activation of secretion of immunosuppressive factors, including interleukin-10 (IL-10), tumor growth factor- β1 (TGF-β1) and, cytotoxic T-lymphocyte protein (CTLA-4) [17]. Finally, ExMVs may stimulate MDCs by miRNA activating the expression of c-Myc [34]. The immunosuppressive effect of MDCs could also be enhanced by ExMVs mediating monocyte differentiation into MDCs [34]. Interestingly, in recently published reports, MSCs-derived ExMVs enriched in miRNA222-3p increased the Th1/Th2 ratio and promoted AML cell apoptosis by regulating negative interferon regulator factor-2 (IRF2) expression [57]. In contrast, AML cells-derived ExMVs inhibited the cytotoxicity of NK cells by activating the TGF−β signaling pathway [58]. What is also important to mention is that all these immunosuppressive effects of leukemic ExMVs may create some problems for successful outcomes of immunotherapies based on the application of CAR-T cells, NK-CAR cells, or adoptive transfer of activated NK cells.

Another important aspect is the role of ExMVs in increasing the resistance of leukemic cells to chemotherapy. This is mainly achieved by increasing the resistance of blast cells to apoptosis. One of the mechanisms elucidated in AML cells is ExMVs transferred BMP-2 that activate in leukemic blasts unfolded protein response pathway that, as known, protects malignant cells from chemotherapy [59]. There are also other examples of increasing resistance of leukemia cells to chemotherapy, as seen e.g., in the case of etoposide. The major mechanism of action of etoposides is by inhibiting topoisomerase II activity, resulting in large-scale replisome collapse and compromising genomic integrity, leading to the production of DNA breaks and eliciting a response that affects several aspects of cell metabolism [60]. However, as shown in the case of multiple myeloma (MM), ExMVs derived from this malignancy upregulate in stroma cells interleukin-8 (IL-8) that convey resistance of MM cells to etoposide [61]. On the other hand, as discussed in a previous paragraph in some of the reports, ExMVs derived from MSCs may have opposite effects by promoting apoptosis of leukemic cells and thus facilitating their elimination by chemotherapy. It is another example of how difficult it is sometimes to dissect and predict the biological pleiotropic effects of ExMVs.

## ExMVs as a biomarker of hematopoietic malignancies

In contrast to some solid tumors, as of today, there are no reliable surface markers specific for leukemic ExMVs. This is because leukemia and hematopoietic cells share several common antigens and receptors. However, some promising molecular signatures of their cargo have been identified. Accordingly, some microRNA species’ expression patterns (e.g., miR-10b and miR-125b) could be correlated with a worse disease prognosis [24, 25, 45]. Similarly, as biomarkers could serve the expression of some long non-coding RNAs, for example, in the case of AML cells LINC00265, LINC00467, or UCA1 [25, 45]. The sensitivity of detection and better identification of potential biomarkers at the RNA level will be improved by employing sensitive next-generation RNA sequencing or proteomics on highly purified ExMVs. Nevertheless, more research needs to be done on early detection of the dysregulation of ExMVs “cargo molecules” that may contain oncogenic transcripts and mRNA species related to basic function of leukemic cells [39–41].

## The inhibition of secretion, uptake, and removal from circulation of ExMVs – current status and potential problems

The unwanted effects of ExMVs lead to the development of several strategies aimed at (i) their removal from circulation, (ii) inhibition of secretion, and (iii) interference with their uptake by target cells [8, 17, 22]. These strategies were mainly developed for solid malignancies but also have potential applications for leukemia. ExMVs could be removed from the circulation by plasmapheresis combined with columns decorated with antibodies against some of their surface antigens. A potential target, for example, would be the application of antibodies against immune checkpoint proteins expressed by some ExMVs, e.g., PD-L1, which is described at ExMVs and inhibits immune response against leukemia cells [54, 55]. Elimination of such unwanted ExMVs would be beneficial during therapy.

On the other hand, inhibition of ExMVs secretion could be achieved by some (i) drugs such as dimethyl amiloride, proton pump inhibitors, or neutral sphingomyelinase-2 inhibitors, (ii) silencing RAB27 family of proteins that are mediators of their secretion, and, finally (iii) targeting endosomal sorting complexes required for transport (ESCRT) proteins and some GTPases involved in production of ExMVs [8, 17]. Finally, the uptake of ExMVs could be perturbed by employing heparin or blocking phosphatidylserine on their surface by Annexin V or diannexin [8, 17]. All these strategies, however, must be evaluated by weighing up potential benefits and losses as they also target ExMVs that could be beneficial for anti-leukemic response or may affect some ExMVs-mediated vital pathways in the normal cells. Therefore, this approach requires further studies and, most importantly, developing alternative, more leukemia ExMVs-targeted strategies.

## Therapeutic application of ExMVs for drug delivery in hematological malignancies

Taking advantage of the fact that ExMVs derived from normal cells may have advantages over synthetic liposomes or nanoparticles and may well protect their inside cargo by a membrane bilayer, they could be harnessed for the delivery of some drugs during chemotherapy or genes employed in gene therapies [25, 32]. ExMVs for therapeutic applications may be generated from normal cells, e.g., from established in vitro BM stroma or endothelial cell cultures [8] or even derived from the cultures of induced pluripotent cell lines (IPs) [62]. So far, applying ExMVs from IPs is the safest application of these cells in therapy. One of the therapeutic advantages of ExMVs is that, because of their small size, they can penetrate the blood-brain barrier and target leukemic cells infiltrating the central nervous system [63].

First clinical trials with ExMVs encapsulating anti-leukemia drugs, such as imatinib, paclitaxel, and doxorubicin, or the anti-inflammatory compound curcumin, have been initiated [22, 32, 33]. Moreover, as recently reported, engineered MSCs-derived ExMVs loaded with miR-34-5p were highly influential in eradicating AML cells in humanized AML mouse models [64]. These particular ExMVs were engineered to overexpress the fused protein lysosome-associated membrane protein 2-interleukin 3 (Lamp2b-IL3) and hematopoietic cell E-selectin/L-selectin ligand (HCELL) to target leukemic cells better [64].

Nevertheless, considering the clinical application of ExMVs to treat leukemia, some things could be improved, including (i) optimization of isolation protocols and (ii) tracking their delivery in vivo. We must standardize clinical-grade production protocols that comply with GMP requirements [65–67]. The methods employed so far for their isolation and purification are based on initial centrifugation of collected cell supernatants to remove cell debris, followed by ultracentrifugation of cell-free extracts on density gradients, size exclusion chromatography, filtration, precipitation, the use of magnetic or agarose beads, and combinatory approaches using these methods. Despite some progress in the field, most isolation/purification strategies are still under development for potential clinical use.

Nevertheless, while promoting the therapeutic application of ExMVs in the clinic, we must be alert to potential “off-target” side effects of such therapies. In vivo, the application of ExMVs bears the risk of hypercoagulation, acute fibrinolysis, and the development of cardiovascular complications [68]. The induction of coagulation is a feature of both normal platelet ExMVs and leukemic ExMvs, as both may express tissue factor. Recent discovery of intracellular complement (complosome) in hematopoietic cells including HSCs raises a question if ExMVs may transfer active complement proteins to other cells [69]. Moreover, in the case of ExMVs derived from induced pluripotent stem cells (iPS), there is a danger of cargo transfer promoting the neoplastic transformation of target cells [70]. Despite some problems, the therapeutic application of engineered ExMVs in treating malignancies has a future.

## Conclusions

Evidence has accumulated that leukemia cells are growing in microenvironments enriched for ExMVs derived from malignant blasts and generated in response to tumor cells from cells residing in hematopoietic organs. Further research is needed to decipher better their molecular signatures comprised of mRNA species (mRNA, miRNA, and long noncoding RNA), proteins, bioactive lipids, and signaling nucleotides (eATP, adenosine) in both ExMVs released by leukemic cells and cells in hematopoietic microenvironment. We are still looking to identify diseases-specific signatures of ExMVs that could employed in diagnosis and in tracking response to therapy. We also need to evaluate the effect of sex, age, and clinical stages of malignancies on their molecular composition. It is necessary to optimize rapid and standardized methods for isolating ExMVs, measuring their number, and purifying them efficiently from biological fluids. If we consider their application as drug vesicles in therapies, developing first isolation protocols corroborating with GMP standards is essential. We still need to identify better signals that promote the release of ExMVs from leukemic and normal cells and strategies to control their release. Thinking about their therapeutic application in the clinic, we also have to consider potential “off-target” side effects of such therapy, including, for example, the risk of hypercoagulation or, in the case of ExMVs derived from induced pluripotent stem cells, the possibility of transfer of cargo that may potentially promote neoplastic transformation of target cells. We will continue to see progress, particularly with new therapeutic and diagnostic applications of ExMVs. Therefore, let us stay alert to exciting new developments!
